# Sildenafil (Viagra) sensitizes prostate cancer cells to doxorubicin-mediated apoptosis through CD95

**DOI:** 10.18632/oncotarget.6749

**Published:** 2015-12-24

**Authors:** Anindita Das, David Durrant, Clint Mitchell, Paul Dent, Surinder K. Batra, Rakesh C. Kukreja

**Affiliations:** ^1^ Department of Internal Medicine, Pauley Heart Center, Virginia Commonwealth University, Richmond, VA, USA; ^2^ Department of Biochemistry and Molecular Biology, Virginia Commonwealth University, Richmond, VA, USA; ^3^ Department of Biochemistry and Molecular Biology, Buffett Cancer Center, University of Nebraska Medical Center, Omaha, NE, USA

**Keywords:** PDE5, doxorubicin, CD95, FLIP, prostate cancer

## Abstract

We previously reported that Sildenafil enhances apoptosis and antitumor efficacy of doxorubicin (DOX) while attenuating its cardiotoxic effect in prostate cancer. In the present study, we investigated the mechanism by which sildenafil sensitizes DOX in killing of prostate cancer (PCa) cells, DU145. The death receptor Fas (APO-1 or CD95) induces apoptosis in many carcinoma cells, which is negatively regulated by anti-apoptotic molecules such as FLIP (Fas-associated death domain (FADD) interleukin-1-converting enzyme (FLICE)-like inhibitory protein). Co-treatment of PCa cells with sildenafil and DOX for 48 hours showed reduced expression of both long and short forms of FLIP (FLIP-_L_ and -_S_) as compared to individual drug treatment. Over-expression of FLIP-s with an adenoviral vector attentuated the enhanced cell-killing effect of DOX and sildenafil. Colony formation assays also confirmed that FLIP-_S_ over-expression inhibited the DOX and sildenafil-induced synergistic killing effect as compared to the cells infected with an empty vector. Moreover, siRNA knock-down of CD95 abolished the effect of sildenafil in enhancing DOX lethality in cells, but had no effect on cell killing after treatment with a single agent. Sildenafil co-treatment with DOX inhibited DOX-induced NF-κB activity by reducing phosphorylation of IκB and nuclear translocation of the p65 subunit, in addition to down regulation of FAP-1 (Fas associated phosphatase-1, a known inhibitor of CD95-mediated apoptosis) expression. This data provides evidence that the CD95 is a key regulator of sildenafil and DOX mediated enhanced cell death in prostate cancer.

## INTRODUCTION

Sildenafil citrate (Viagra), a highly selective inhibitor of cGMP-specific phosphodiesterase type 5 (PDE5), is used clinically for treating erectile dysfunction (ED) and pulmonary hypertension. Several studies have shown that PDE5 expression is increased in multiple human carcinomas including metastatic breast cancers, colon adenocarcinoma, bladder squamous carcinoma, and lung cancers as compared to adjacent normal tissues [1–6], suggesting its potential role in controlling tumor cell growth and death. PDE5 was also detected as a predominant isoform of cGMP-PDEs in many carcinoma cells lines in culture, including colonic adenocarcinoma (SW480, HCT116, HT29, T84), breast cancer (HTB-26, MCF-7), lung cancer, bladder and prostate cancer (LNCAP, PC-3), and leukemia [6–8]. All forms of prostate cancer therapy cause significant risk of ED due to trauma sustained by the cavernosal nerves [9]. PDE5 inhibitors have been shown to improve erectile function post-radical prostatectomy [10–13]. Our laboratory first demonstrated that co-treatment with sildenafil potentiates antitumor efficacy of doxorubicin (DOX) in prostate cancer, which was mediated by enhanced generation of ROS, up-regulation of caspase-3 and caspase-9 activities, reduced expression of Bcl-xL, and phosphorylation of Bad [14]. Sildenafil also potentiated DOX-induced killing of androgen independent human prostate cancer cells and inhibited tumor growth in mice bearing prostate tumor xenografts [14].

Despite its clinical efficacy, the use of DOX is limited by a dose-dependent delayed and progressive cardiomyopathy often observed several years after cessation of treatment [15;16]. A great deal of effort has been expended in preventing or mitigating the cardiotoxic side effects of DOX without reducing the antitumor efficacy or causing additional toxic effects. Our study in mice bearing prostate tumor xenografts also confirmed that sildenafil and DOX combination ameliorated DOX-induced cardiac dysfunction, which is consistent with our previous study showing improved left ventricular (LV) function with PDE5 inhibitors in DOX-treated mice [17;18].

Recent studies suggest that Fas/Apo-1/CD95, a member of the tumor necrosis factor (TNF) receptor superfamily, is a potential anti-cancer factor as it can induce apoptosis in tumor cells [19]. Prior studies from our laboratories have demonstrated that sildenafil enhances the cytotoxicities of multiple well-established chemotherapeutic drugs [14;20-22]. Sildenafil potentiated chemotherapy killing through activation of the CD95 death receptor pathway, generation of reactive oxygen species, and mitochondrial dysfunction in gastrointestinal/genitourinary cancers, hepatoma, colorectal cancer, glioblastoma, medulloblastoma cells and breast cancer cells [20–22]. However, the role of CD95 in sildenafil-induced enhanced toxicity of DOX in prostate cancer is not known. In addition, despite the fact that Fas/CD95 is expressed in many cancer cells, some tumors, such as prostate cancer, display resistance to Fas-induce apoptosis due to the decreased expression of Fas in a large fraction of prostate cancer [23]. By contrast, the expression of FLIP (FLICE-like inhibitory protein), an inhibitor of Fas-mediated apoptosis, was strong in most cases of prostate cancer [23]. Therefore, in this study, we attempted to identify the central mechanism of sildenafil-induced enhanced chemotherapeutic efficacy of DOX in prostate cancer. We hypothesized that CD95/FLIP may be the key regulators in sildenafil and DOX mediated killing of prostate cancer cells.

## RESULTS

### Effect of sildenafil and DOX on the expression of FLIP

**Figure 1:**
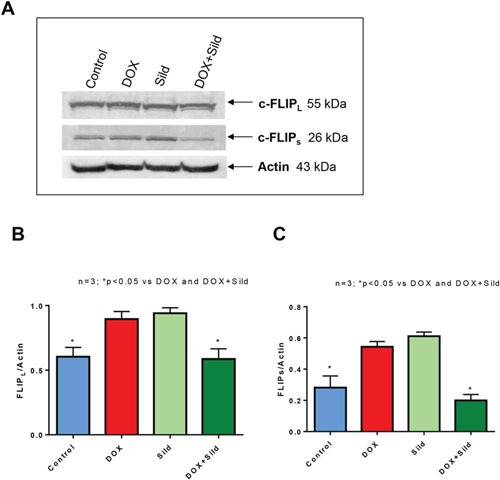
Effect of sildenafil and DOX on FLIP-_L_ and FLIP-s expression in DU 145 cells **(A)** Representative immunoblots for FLIP-_L_, FLIP-s and Actin in DU145 cells after 48 hr of treatment with DOX (0.5 μM) and/or Sild (10 μM). Densitometry analysis of the ratios of **(B)** FLIP-_L_ to Actin, and **(C)** FLIP-_S_ to Actin. Abbreviations: doxorubicin – DOX; Sildenafil – Sild.

**Figure 2:**
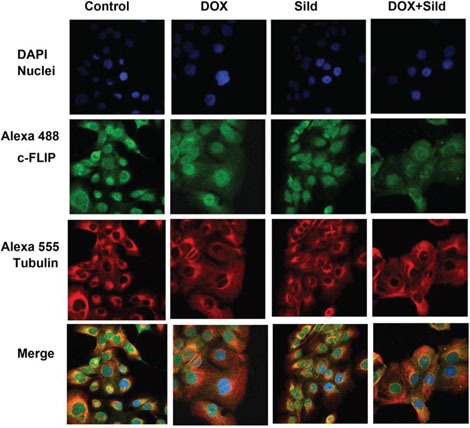
Sildenafil and DOX co-treatment reduced FLIP expression in DU145 cells Representative images of the immunohistochemical staining for FLIP in DU145 cells following 48 hr treatment with DOX (0.5 μM) and/or Sild (10 μM). The paraformaldehyde fixed cells were incubated with rabbit FLIP-s and mouse tubulin antibodies and visualized by Alexa Fluor 488-conjugated anti-rabbit IgG (green fluorescence) and Alexa 555-conjugated anti-mouse IgG (red fluorescence), then examined by confocal microscopy. Abbreviations: doxorubicin – DOX; Sildenafil – Sild.

As shown in Figure 1, treatment with DOX or sildenafil alone did not alter the expression of FLIP-_L_ or –s as compared to control. However, co-treatment with DOX and sildenafil reduced the expression of both FLIP-_L_ and -_S_ compared to control and DOX alone. Immunohistochemistry also confirmed the reduction of the expression of FLIP following sildenafil and DOX co-treatment (Figure 2).

### Role of FLIP in synergistic killing effect of sildenafil and DOX co-treatment

**Figure 3:**
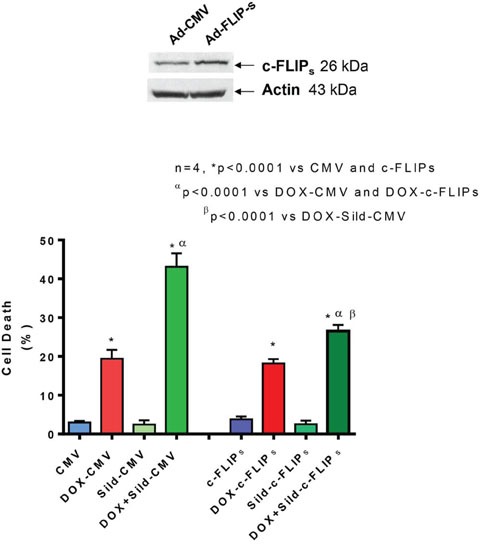
Overexpression of FLIP-s abolished synergistic effect of sildenafil and DOX co-treatment on DU145 cells Upper panel shows overexpression of FLIP-s by adenoviral construct. Bar chart represents cell death following overexpression of FLIP-s or empty vector and 24 hr treatment with DOX (0.5 μM) and/or Sild (10 μM). Abbreviations: doxorubicin – DOX; Sildenafil – Sild.

FLIP-s was overexpressed in DU145 cells by adenoviral infection (Figure 3 upper panel). cell death assessed by trypan blue exclusion assay confirmed that sildenafil potentiated DOX-induced cell killing in cells infected with control virus (Ad-CMV). The overexpression of FLIP-s inhibited the additive cell killing effect of sildenafil as compared to DOX alone (Figure 3). DOX-induced cell death was increased with sildenafil co-treatment from 19.4±2.3% to 43.1±3.5% in adeno-CMV infected cells (P<0.05). In cells overexpressing FLIP-s, sildenafil co-treatment also increased DOX-induced cell death from 18.2±1.1% to 26.5±1.6% although said increase was not as high as adeno-CMV infected cells. Colony formation assays performed using median dose effect isobologram analysis also showed the overexpression of FLIP-s inhibited the DOX and sildenafil-induced synergistic killing of DU145 cells (Table 1) with CI >1.

**Table 1:** Overexpression of c-FLIP_-s_ blocks synergistic killing effect of Sildenafil and DOX co-treatment in DU145 cells

	DOX (μM)	Sildenafil (μM)	Fraction Affected	CI
Ad-CMV	0.5 1.0 1.5	5 10 15	0.28 0.55 0.63	0.50 0.52 0.64
Ad-c-FLIP_S_	0.5 1.0 1.5	5 10 15	0.02 0.04 0.11	1.83 2.30 1.71

DU145 cells were plated as single cells (250–1,500 cells/well) in sextuplicate, which were then infected with an Ad.CMV or Ad.c-FLIPs adenovirus 12 hr following plating. 24 hr after infection, cells were treated with doxorubicin (DOX, 0.5–1.5 μM), sildenafil (5–15 uM), or with both drugs combined, as indicated at a fixed concentration ratio to perform median dose effect analyses for the determination of synergy. After drug exposure (24 hr), the media was changed and cells cultured in drug free media for an additional 10–14 days. Cells were fixed, stained with crystal violet, and colonies of >50 cells/colony counted. Colony formation data were entered into the Calcusyn program and combination index (CI) and Fraction Affected (Fa) values determined. A CI value of less than 1.00 indicates synergy.

### CD95 knock-down abolishes the enhanced cell killing effect of DOX and sildenafil co-treatment

**Figure 4:**
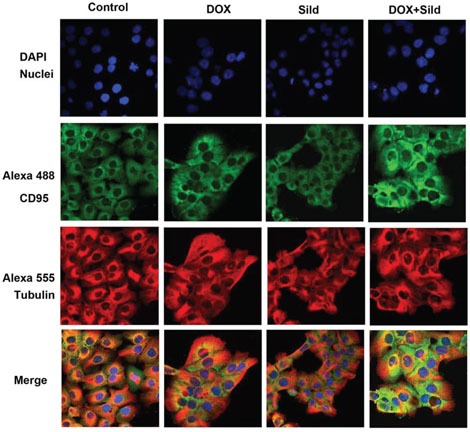
Sildenafil and DOX co-treatment enhanced surface localization of CD95 in DU 145 cells Representative images of the immunohistochemical staining for CD95 in DU 145 cells following 48 hr treatment with DOX (0.5 μM) and/or Sild (10 μM). The paraformaldehyde fixed cells were incubated with rabbit CD95 and mouse tubulin antibodies and visualized by Alexa Fluor 488-conjugated anti-rabbit IgG (green fluorescence) and Alexa 555-conjugated anti-mouse IgG (red fluorescence), then examined by confocal microscopy. Abbreviations: doxorubicin – DOX; Sildenafil – Sild.

**Figure 5:**
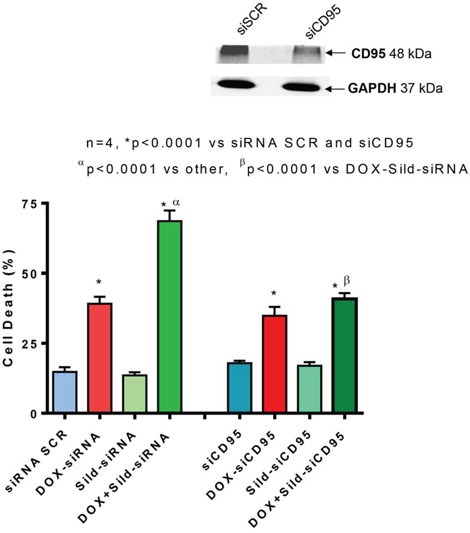
Knock-down of CD95 abolishes sildenafil and DOX-induced toxicity in DU145 cells Upper panel shows knock-down of CD95 by siRNAs. Bar chart represents cell death following knock-down of CD95 with siRNA or scramble RNA (siSCR as control) and 24 hr treatment with DOX (0.5 μM) and/or Sild (10 μM). Abbreviations: doxorubicin – DOX; Sildenafil – Sild.

Immunohistochemistry showed enhanced cell surface localization of CD95 protein in DU145 cells following DOX treatment (Figure 4). Sildenafil co-treatment further enhanced the cell surface localization of CD95. Since the loss of CD95 expression is common in tumors from patients with metastatic/advanced cancer, we examined whether sildenafil could enhance DOX-toxicity in DU145 cells with reduced CD95 function. Western blot confirmed the knock-down of CD95 expression in DU145 cells by siRNA CD95 (Figure 5 upper panel). Cell death assessed by trypan blue exclusion assay confirmed that sildenafil potentiated DOX-induced cell killing in scramble siRNA transfected DU145 cells; however, knock-down of CD95 abolished the simulated cell killing effect of sildenafil compared to DOX alone (Figure 5). DOX-induced cell death was increased with sildenafil co-treatment from 39.2±2.4 to 68.6±3.8% in scramble siRNA transfected cells (P<0.05). Sildenafil co-treatment did not significantly increase DOX-induced cell death in DU145 cells transfected with siRNA CD95, i.e., from 34.8±3.2% to 40.9±2.0%.

### Sildenafil and DOX co-treatment stimulates co-localization of caspase-8 with CD95 in DISC

**Figure 6:**
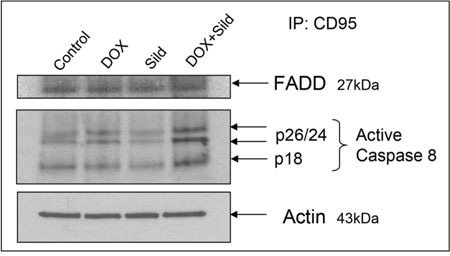
Sildenafil and DOX co-treatment increases recruitment of FADD with CD95 in DISC formation and activates caspase-8 Following 48 hr of treatment with Sild (10 μM) and/or DOX (0.5 μM), total protein of DU145 cell was subjected to immunoprecipitated with rabbit CD95 antibody. The amount of co-immunoprecipitating caspase 8 was determined after SDS-PAGE and Western blotting with mouse caspase 8 (active format) and FADD antibodies. Abbreviations: doxorubicin – DOX; Sildenafil – Sild.

Immunoprecipitation with a CD95 antibody and immunoblotting with an active caspase-8 antibody showed showed an increase in the active form of caspase-8 (p26/24 and p18) after DOX treatment as compared to control and sildenafil alone (Figure 6). Sildenafil co-treatment with DOX further stimulated the active form of caspase-8 with enhanced recruitment of FADD with CD95 in DISC formation (Figure 6).

### Sildenafil and DOX co-treatment reduces FAP-1 expression and inhibits NFκB

**Figure 7:**
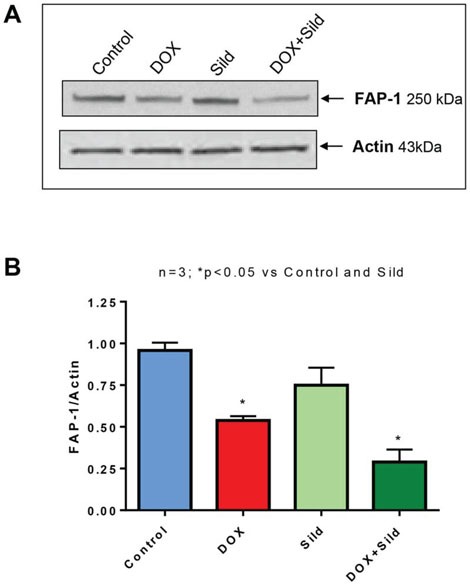
Sildenafil and DOX co-treatment reduces FAP-1 expression in DU 145 cells **(A)** Representative immunoblots for FAP-1 and Actin in DU145 cells after 48 hr of treatment with DOX (0.5 μM) and/or Sild (10 μM). **(B)** Densitometry analysis of the ratios of FAP-1 to Actin. Abbreviations: doxorubicin – DOX; Sildenafil – Sild.

**Figure 8:**
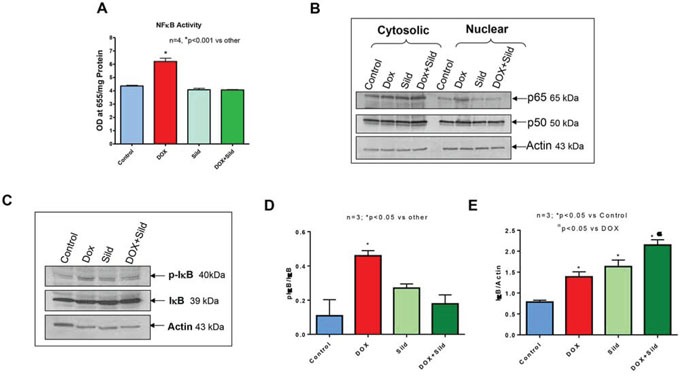
Sildenafil and DOX co-treatment reduces NF-κB activity in DU145 cells **(A)** NFκB activity in DU145 cells following 48 hr of treatment with DOX (0.5 μM) and/or Sild (10 μM). **(B)** Subcellular localization of p65 and p50 after 48 hr of treatment with DOX (0.5 μM) and/or Sild (10 μM). **(C)** Phosphorylation of IκB after 48 hr of treatment with DOX (0.5 μM) and/or Sild (10 μM). Densitometry analysis of the ratios of **(D)** pIκB to IκB, and **(E)** IκB to Actin. Abbreviations: doxorubicin – DOX; Sildenafil – Sild.

Treatment with sildenafil and DOX caused reduced expression of FAP-1 as compared to controls i.e., non-treated, sildenafil or DOX (Figure 7). Treatment with DOX significantly increased NFκB activity compared to control and sildenafil alone (Figure 8A). However, sildenafil co-treatment with DOX significantly reduced NFκB activity as compared to DOX alone. DOX also induced the nuclear translocation of the p65 subunit from cytosol compared to control (Figure 8B). Sildenafil co-treatment with DOX reduced p65 nuclear translocation as compared to DOX alone. Moreover, nuclear translocation of p50 was slightly enhanced by DOX, which was blocked with sildenafil co-treatment. Phosphorylation of IκB was also induced with DOX treatment which was reduced by sildenafil co-treatment (Figure 8C).

## DISCUSSION

The death receptor Fas (APO-1/CD95) induces apoptosis in many tissues upon cross-linking by its ligand (FasL), but a number of cancer cells exhibit resistance to Fas induced apoptosis. In fact, prostate cancer is resistant to Fas-mediated apoptosis despite high levels of Fas surface expression and no mutation in the Fas gene [24–26]. Suppression of apoptotic signaling seems to be one of the hallmarks of cancer that is regulated by specific genetic and epigenetic mechanisms, including the down-regulation of Fas death receptor [27;28]. Therefore, it is clinically important to understand the underlying mechanisms by which cancer cells acquire such resistance. Our previous study demonstrated a potential utility of sildenafil in enhancing the chemotherapeutic efficacy of DOX in prostate cancer while simultaneously providing cardioprotective benefits [14]. In bladder cancer cells, as well as pancreatic cancer cells, we showed that sildenafil enhanced the lethality of mitomycin C, doxorubicin, cisplatin, and gemcitabine via activation of CD95 [20]. In the present study, we further investigated the molecular events of CD95-induced apoptosis in prostate cancer cells following combination therapy with DOX and sildenafil. Our results show that CD95 plays a critical role in enhancing apoptosis by combination treatment with sildenafil and DOX in prostate cancer cells, DU145.

One major regulator of CD95-mediated apoptosis at the DISC level is c-FLIP [29]. Multiple splice variants of c-FLIP have been reported but so far only a 26 kDa short form (c-FLIP_s_), a 24 kDa form (c-FLIP_R_) and a 55 kDa long form (c-FLIP_L_) could be detected at protein level [30;31]. The inhibition of apoptosis by c-FLIP_L_ has been shown to be through recruitment to and cleavage in the DISC, because c-FLIP_L_ induces a conformation of procaspase-8 that allows partial but not complete proteolytical processing of the caspase-8 molecule. Recruitment of c-FLIP_S_ to the DISC prevents caspase-8 cleavage completely [32]. Several studies reported that high expression of FLIP promotes tumor growth and facilitates immune escape of tumors [30;33]. Ectopic overexpression of c-FLIP accelerated progression to androgen independent growth by inhibiting apoptosis in LNCaP prostate tumors implanted in nude mice [34]. Down-regulation of FLIP confers not only sensitivity to Fas-induced apoptosis but also to chemotherapy-induced apoptosis in various tumor models [35;36]. In addition, down-regulation of FLIP has been shown to sensitize breast cancer cells to DOX-induced apoptosis [37]. Our prior studies showed that overexpression of BCL-xL and c-FLIP-s suppressed synergetic effect of sildenafil and other chemotherapeutic drugs (e.g. vincristine/etoposide/cisplatin) in medulloblastoma cells [22]. In the present study, we observed that the expression of c-FLIP_L_ and c-FLIP_s_ were reduced in DU145 cells with sildenafil and DOX co-treatment as compared to DOX alone and control. Moreover, overexpression of c-FLIPs blocked the enhanced cell killing effect of sildenafil with DOX co-treatment as compared to DOX alone. A colony forming assay forming assay further confirmed that overexpression of c-FLIP_s_ blocked the synergistic effect of sildenafil and DOX in enhancing cell killing. Our results suggest that the reduction of c-FLIP by combination treatment with sildenafil and DOX could be a potential molecular intervention in induction of apoptotic cell death in prostate cancer.

The level of cell surface expression of CD95 is a critical parameter in determining the capability cells to undergo apoptosis. CD95 tyrosine phosphorylation is an essential step for CD95-activation, subsequent membrane targeting and apoptosis induction [38]. In the present study, CD95 cell surface localization was increased after DOX treatment, and sildenafil co-treatment further enhanced this effect. Moreover, the knock-down of CD95 with siRNA abolished the simulated cell killing effect of sildenafil as compared to DOX alone. Taken together, this data indicates a potential role of CD95 in enhanced cytotoxicity of prostate cancer cells by co-treatment with sildenafil and DOX. These results are consistent with a previous study showing significantly reduced synergistic effect of sildenafil and COX-2 inhibitor celecoxib in human glioma cell with over-expression of c-FLIP-s or knock down of CD95/FADD [21].

CD95 stimulation leads to the formation of death-inducing signaling complex (DISC) on the cell membrane through recruitment of Fas-associated death domain-containing protein (FADD) to the intracellular death domain (DD) of CD95 receptor [39]. In DISC, the death effector domain (DED) of FADD interacts with the N-terminal tandem DEDs of procaspases-8, -10, and c-FLIP, which leads to autoproteolytical cleavage and activation of caspase-8 [40]. In the cleavage steps of procaspase-8, the active enzyme subunits p18, p10, and the prodomains p26/p24 are produced. Finally the active caspase-8 heterotetramer p10_2_-p18_2_ is released into the cytosol to stimulate the apoptotic signal. In the present study, through immunoprecipitation of protein with CD95 and immunoblotting with caspase-8, we confirmed that the active formats of caspase 8 (p26/p24 and p18) are increased after sildenafil co-treatment with DOX compared to control and DOX alone. This data suggests that co-treatment of sildenafil and DOX induces caspase-8 activation by stimulating CD95 in prostate cancer cells.

The protein-tyrosine phosphatase FAP-1/PTP-Bas/PTPN13 interacts with human Fas protein (CD95) and prevents its export from the cytoplasm to the cell surface in cancer cells [41–43]. FAP-1 has been reported to dephosphorylate CD95 on Tyr291- a site important for the receptor to internalize and to signal apoptosis [28;41;44-47] while also rendering cells resistant to CD95-mediated apoptosis [42;48]. FAP-1 overexpression correlates with the resistance of some human malignant cells to Fas-mediated apoptosis [41]. Ivanov et al. (2006) demonstrated that FAP-1 expression is transcriptionally up-regulated by NF-κB, a major antiapoptotic transcription factor that restricts Fas protein trafficking to the cell surface thereby, facilitating the survival of cancer cells [28]. The presence of the putative NF-κB-binding elements are recognized in the promoter region of the FAP-1 gene [49;50]. NF-κB dependent regulation of the Fas gene is also well established [51;52]. It has been reported that some chemotherapeutic agents, including DOX, induce the activation of NF-κB in cancer cells that inhibits apoptosis and promotes cancer growth and are also responsible in part for drug resistance in cancer cells [53–55]. Therefore, targeted inactivation of NF-κB without systemic toxicity in combination with chemotherapeutic agents may increase the efficacy of cancer cell killing. Interestingly, IκBα, an inhibitor of NF-κB, is also a substrate for FAP-1, which dephosphorylates IκBα at Tyr 42, and renders subsequent phosphorylation and its ubiquitination [56], which in turn, allows the p65 subunit translocation to nucleus and NF-κB activation. In head and neck cancer, treatment with phosphatase inhibitor or silencing of FAP-1 abolishes their resistance to apoptosis, suggesting that FAP-1 activity could be responsible for NF-κB activation and resistance of cells to Fas-mediated apoptosis [57].

The present study also showed that sildenafil co-treatment with DOX reduced the expression of FAP-1 in DU145 cells. DOX significantly increased the activity of NF-κB by inducing nuclear translocation of p65 subunit in DU145 cells. However, DOX-induced activation of NF-κB and nuclear translocation of p65 are inhibited following sildenafil co-treatment with DOX. Moreover, IκBα phosphorylation is also reduced which leads to a reduction of nuclear translocation of p65 and p50. Previous studies show that NF-κB transcriptionally up-regulates the expression of c-FLIP, which inhibits caspase-8 activation and the Bcl-2 family of proteins, including Bcl-2, Mcl-1, and Bcl-xL, and counteract the action of pro-apoptotic proteins including Bid, Bad, and Bax the action of pro-apoptotic proteins including Bid, Bad, and Bax [58–60]. Therefore, we suggest that sildenafil abolishes DOX–induced activation of NF-κB, which also down-regulates FAP-1 and c-FLIP and stimulates CD95-mediated apoptosis in prostate cancer cells.

Decreased expression or functional mutation of Fas has been found in various malignant tumors, which impairs apoptotic signal transduction and leads to progression of cancer [61;62]. Several single-nucleotide polymorphisms have been identified in the promoter region of the FAS gene, including the substitution of A to G at position −670 (FAS −670A/G) [63]. A recent meta-analysis study identified a significant association among Fas-670 A/G polymorphism with the risk of prostate cancer as well as the impairment of tumor cells sensitivity to apoptosis signaling [64;65]. The present study identified the essential role of CD95 in enhancing apoptosis by combination therapy with sildenafil and DOX in prostate cancer cells. Therefore, prostate cancer patients with Fas-670 A/G polymorphism may be resistant to this combination therapy.

**Figure 9:**
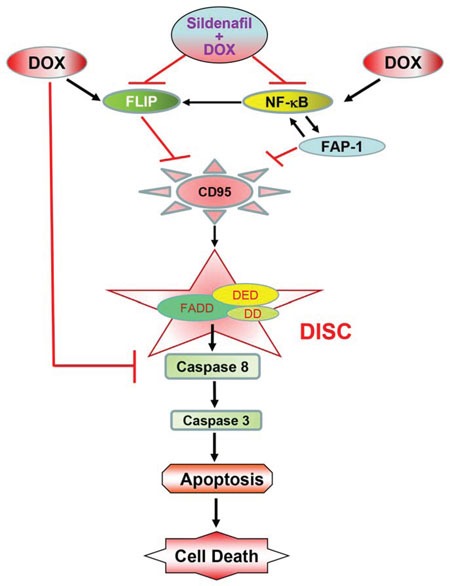
The proposed mechanism by which sildenafil potentiates the cytotoxicity of DOX in prostate cancer Sildenafil co-treatment with doxorubicin (DOX) inhibited DOX-induced enhanced expression of FLIP and activation of NFκB, which led to inhibition of FAP-1, activation of CD95 and the formation of death-inducing signaling complex (DISC) on the cell membrane through recruitment of Fas-associated death domain-containing protein (FADD) to the intracellular death domain (DD) of CD95 receptor. FADD contains another protein–protein interaction domain, termed the death effector domain (DED), which is required for the recruitment of caspases to the DISC. Formation of DISC induces cellular apoptosis by activation of caspase-8 and 3.

In conclusion, the present study has identified a novel mechanism of synergistic cell death with sildenafil and DOX in prostate cancer cells which involves increased surface localization of CD95, with concomitant inactivation of NF-κB and suppression of FLIP and FAP-1 expression (Figure 9). These mechanistic studies may contribute to the expanding use of PDE5 inhibitors in enhancing chemotherapeutic efficacy in prostate cancer, but not in tumors lacking CD95 expression.

## MATERIALS AND METHODS

### Treatment of prostate cancer cells

Human prostate carcinoma (PCa) cells, DU145 (American Type Culture Collection), were grown in Eagle's Minimum Essential Medium with 10 % FBS and 1% penicillin/streptomycin in 5% CO_2_ atmosphere at 37°C. Cell were treated with DOX (0.5 μM) and/or sildenafil (10 μM) in complete growth medium.

### Cell death assay

Cells were treated with DOX and/or sildenafil for 24 hr in 6-well plate. After trypsinization, cell were stained with trypan blue and counted under microscope (Nikon Eclipse Ti-S).

### Knockdown of CD95 and overexpression of FLIP-s

Cells were transfected with scramble RNA (siSCR as control) or siRNA to knockdown CD95 (purchased from Qiagen, Valencia, CA) or infected with adenoviral vector to overexpress FLIP-s (Vector Biolabs, Philadelphia, PA). After knockdown of CD95 or overexpression of FLIP-s (24 hr), cells were treated with sildenaifl and/or DOX.

### Immunofluorescence analysis

Following treatment with DOX and/or sildenafil, cells on slides were fixed in 4% paraformaldehyde. Cells were permeabilized with 0.2% Triton X-100 in PBS. Cells were blocked with 2% goat serum in PBS and incubated with anti-rabbit CD95 or rabbit FLIP-s and mouse tubulin antibodies for 2 hr at RT. Slides were rinsed with PBS and incubated with Alexa 488-conjugated anti-rabbit IgG (Molecular Probes) and Alexa 555-conjugated anti-mouse IgG (Molecular Probes), then examined by Nikon D-Eclipse C1 confocal microscope.

### Synergy of cell killing

The synergy of killing by sildenafil and DOX was analyzed by colony formation assays using median dose effect isobologram analysis [14]. Briefly, DU145 cells were plated as single cells (250–1,500 cells/well) in sextuplicate. The cells were infected with an Ad.CMV or Ad.c-FLIPs adenovirus after 18 hours of plating. After 24 hours, cells were treated with doxorubicin (DOX, 0.5–1.5 μM), or/with sildenafil (5–15 uM), at a fixed concentration ratio to perform median dose effect analyses for the determination of synergy. After drug exposure (24 h), the media was changed and cells cultured in drug free media for an additional 10–14 days. Cells were fixed, stained with crystal violet, and colonies of >50 cells/colony counted. Colony formation data were entered into the Calcusyn program and combination index (CI) and Fraction Affected (Fa) values determined. A CI value of less than 1.00 indicates synergy.

### Western blot analysis

Total soluble protein was extracted from treated cells with cell lysis buffer (Cell Signaling Technology, Danvers, MA). The homogenate was centrifuged at 14,000 g for 15 min under 4°C and the supernatant was recovered. Fifty micrograms of protein from each sample were separated by SDS/PAGE and transferred on to a nitrocellulose membrane. The membrane was incubated with various primary antibodies against different proteins (CD95, FLIP, FAP-1, pIκB or IκB, Cell Signaling Technology, Danvers MA, or Actin, Santa Cruz Biotechnology, Inc. Dallas, TX) at a dilution of 1:1,000. The membrane was washed and incubated with horseradish peroxidase-conjugated secondary antibody (1:2,000 dilutions). The blots were developed using a chemiluminescent system (Amersham ECL Plus; GE Healthcare Bio-Sciences Pittsburgh, PA). Subcellular localization of p65 and p50 were determined by separating nuclear and cytoplasmic proteins from DU145 cells after 48 h of treatment with DOX (0.5 μM) and/or Sild (10 μM) using nuclear extraction kits (Abcam, MA). Proteins were then separated by SDS/PAGE and transferred on to a nitrocellulose membrane. The membrane was incubated with p65 and p50 antibodies (Cell Signaling Technology, Danvers MA).

### Immunoprecipitation

Total protein was extracted from treated cells with RIPA buffer. The homogenate was centrifuged at 14,000 g for 15 min under 4°C and the supernatant was recovered. Total protein (400 μg) was subjected to immunoprecipitation by a rabbit CD95 antibody. The amount of co-immunoprecipitating caspase-8 was determined after SDS-PAGE and Western blotting with mouse caspase-8 (active format) and FADD antibodies (Cell Signaling Technology, Danvers MA)

### NF-κB activity assay

Nuclear and cytoplasmic proteins were isolated using nuclear extraction kits (Abcam, MA). Activity of NF-kB was measured using NFkB p65 Transcription Factor Assay Kit Colorimetric kit (Abcam, MA) according to manufacturer's protocol.

### Data analysis and statistics

Data are presented as mean±S.E. The differences between groups were analyzed with one-way analysis of variance following by Student-Newman-Keuls post hoc for pair-wise comparison. P<0.05 was considered to be statistically significant.
